# A Rare Type of Bullous Disorder in a Child. Can It Be Chronic Bullous Disease of Childhood?

**DOI:** 10.1002/ccr3.71746

**Published:** 2025-12-28

**Authors:** Rajeev Yadav, Indira Poudel, Yogesh Poudyal, Nagendra Chaudhary

**Affiliations:** ^1^ Department of Dermatology and Venereology Universal College of Medical Sciences Bhairahawa Nepal; ^2^ Sarnath Skin Centre Bhairahawa Nepal; ^3^ Department of Pediatrics Universal College of Medical Sciences Bhairahawa Nepal

**Keywords:** bullous dermatosis, chronic bullous disease of childhood, immuno‐bullous disease, linear IgA dermatosis, ring of pearls

## Abstract

Chronic bullous disease of childhood (CBDC), also known as childhood linear IgA bullous dermatosis, is a rare autoimmune disorder characterized by sub‐epidermal blistering and linear deposition of immunoglobulin A at the demo‐epidermal junction. Clinical manifestations in children include tense bulla (arranged annularly in the form of ring of pearl) and vesicles over the erythematous skin. Diagnosis is confirmed by skin biopsy from lesions showing sub‐epidermal blisters in histopathological examination and linear deposition of IgA at the dermal‐epidermal junction in direct immunofluorescence. Here, we report a case of CBDC in a 5‐year‐old child who was treated successfully with oral dapsone showing improvement in the skin lesions.

## Key Clinical Message

Chronic bullous disease of childhood (CBDC), also known as childhood linear IgA bullous dermatosis, is a rare autoimmune disorder characterized by sub‐epidermal blistering and linear deposition of immunoglobulin A at the dermo‐epidermal junction. Here, we report a case of CBDC in a 5‐year‐old child who was treated successfully with oral dapsone.

## 1 Introduction

Linear IgA bullous dermatosis (LABD) is a rare autoimmune blistering disease characterized by sub‐epidermal blistering with linear deposition of IgA at the dermal‐epidermal junction both in adults (adult LABD) and children (childhood LABD) [1, 2]. Childhood LABD is also known as chronic bullous disease of childhood (CBDC) which generally occurs in infants and children with tense bulla and vesicles over the erythematous skin where bullae are frequently arranged annularly in the form of “ring of pearls” or “clusters of jewels” [3]. Diagnosis is confirmed with histopathological examination of skin biopsy showing sub‐epidermal blisters and linear deposition of IgA at the dermal‐epidermal junction [4].

Here, we report a case of a 5‐year‐old male child who presented with numerous tense bulla, vesicles, and erosion over the trunk and extremities diagnosed as CBDC, both clinically and histopathologically. The child in this case was treated successfully with oral dapsone, with improvement in the skin lesions.

## 2 Case History and Examination

**FIGURE 1:**
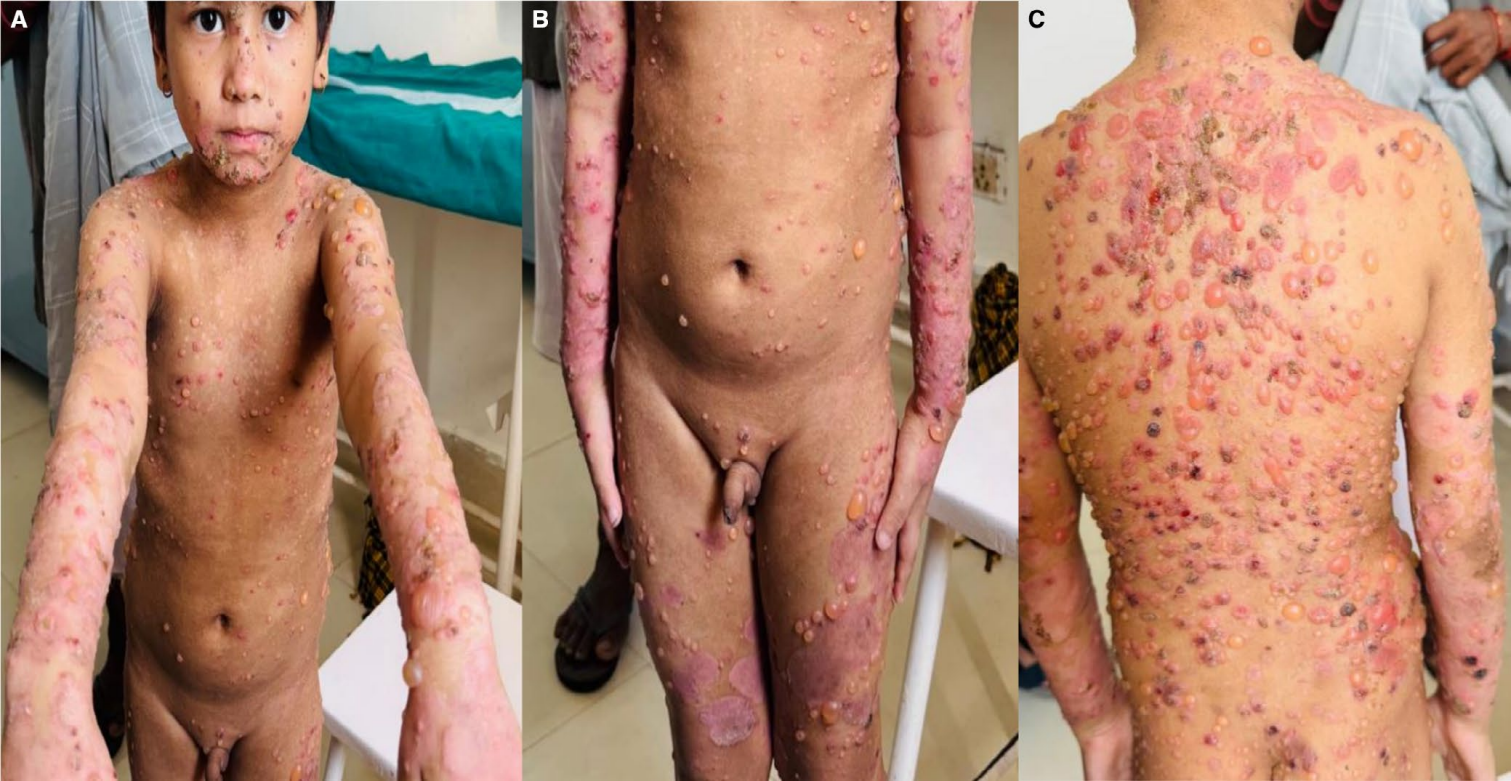
(A, B and C) Multiple, grouped vesicles with bullae in a “string of pearls” or “cluster of jewels” arrangement on the face, chest, hands, legs and back.

A 5‐year‐old male child, born to non‐consanguineous parents, was brought to the department of Dermatology and Venereology at Universal College of Medical Sciences‐ Teaching Hospital (UCMS‐TH), Bhairahawa, Nepal with complaints of numerous tense bulla and vesicles (itchy and painful) predominately over the trunk for 1 month. The skin lesions were also seen on the face, scalp, and extremities, both upper and lower with symmetrical distribution as shown in Figure 1 involving approximately 30% of the body surface area. The child was developmentally normal with no similar history in the siblings. The parents did not give any history of drug intake prior to the onset of bulla.

On clinical examination, the bulla was present over the erythematous base. Some bullae were arranged annularly in “cluster of jewels” or “ring of pearl pattern” with a few hemorrhagic bullae having erosions and crusting. Nikolsky's and Asboe Hansen sign were negative. The involvement of palm, sole, eye, oral mucosa, and genital mucosa was not seen. Other systemic examinations were normal.

## 3 Differential Diagnosis, Investigations and Treatment

**FIGURE 2:**
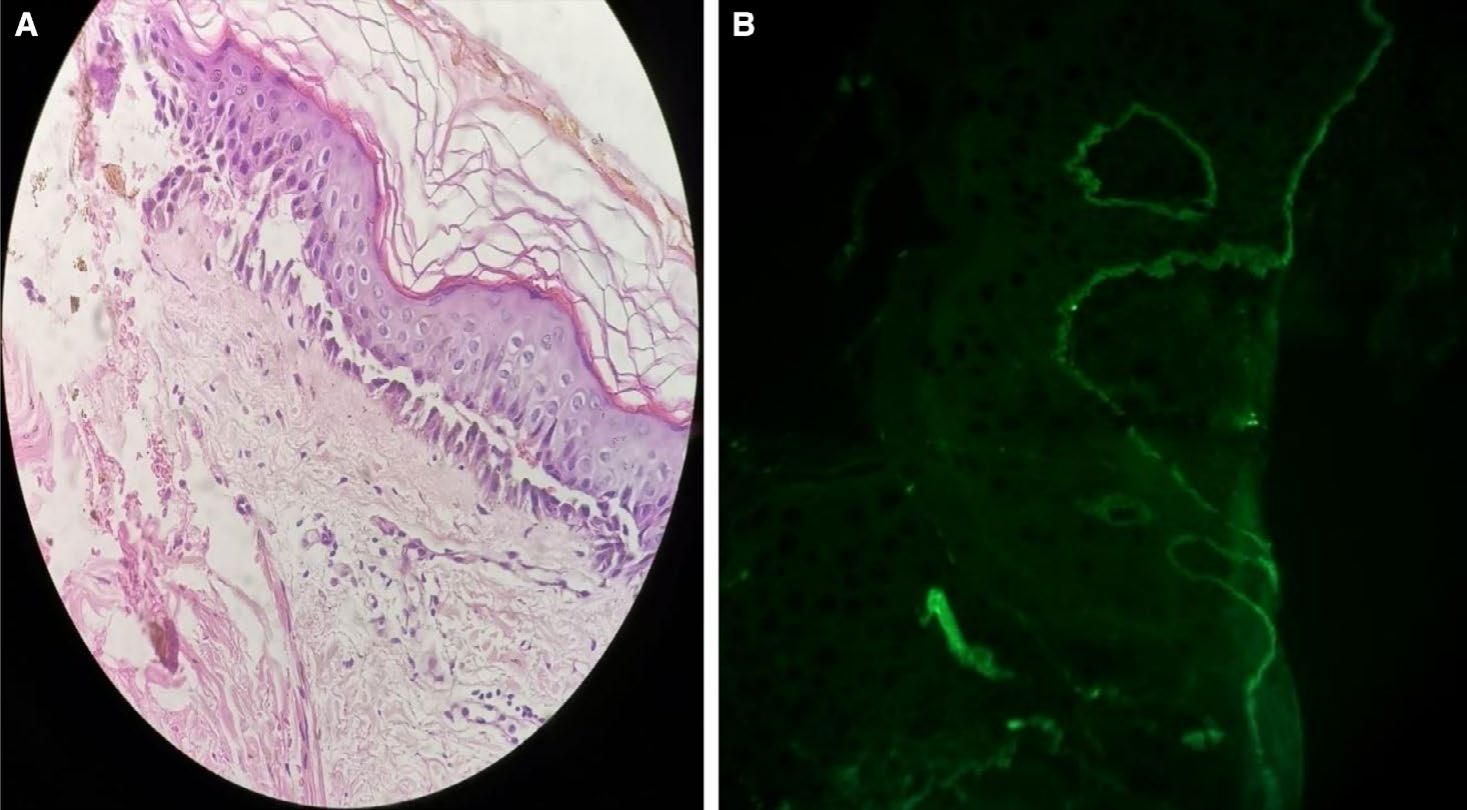
(A) Histopathological examination showing sub‐epidermal separation with neutrophilic infiltrates in the dermis (H and E, 40×); (B) Direct immunofluorescence showing linear deposition of IgA along the sub‐epidermal layer.

Differentials diagnoses of bullous pemphigoid, bullous impetigo, and CBDC were considered. Tzanck smear done from the base of the lesion was negative for multinucleated giant cells and acantholytic cells. Histopathological examination (HPE) showed sub‐epidermal splitting with neutrophilic infiltrate at the dermo‐epidermal junction (Figure 2A).

Direct immunofluorescence (DIF) showed linear deposition of IgA and C3 at the dermo‐epidermal junction (Figure 2B). Complete blood count, renal function test, and liver function test were normal. Initially, the child was treated with systemic steroid (oral prednisolone, 1 mg/kg/day) awaiting glucose‐6‐phosphate dehydrogenase (G6PD) activity report. Later on, oral dapsone (0.5 mg/kg) was added once G6PD activity was adequate and the steroid was tapered gradually. Skin lesions significantly improved within 5 days of starting dapsone.

## 4 Outcome and Follow‐Up

**FIGURE 3:**
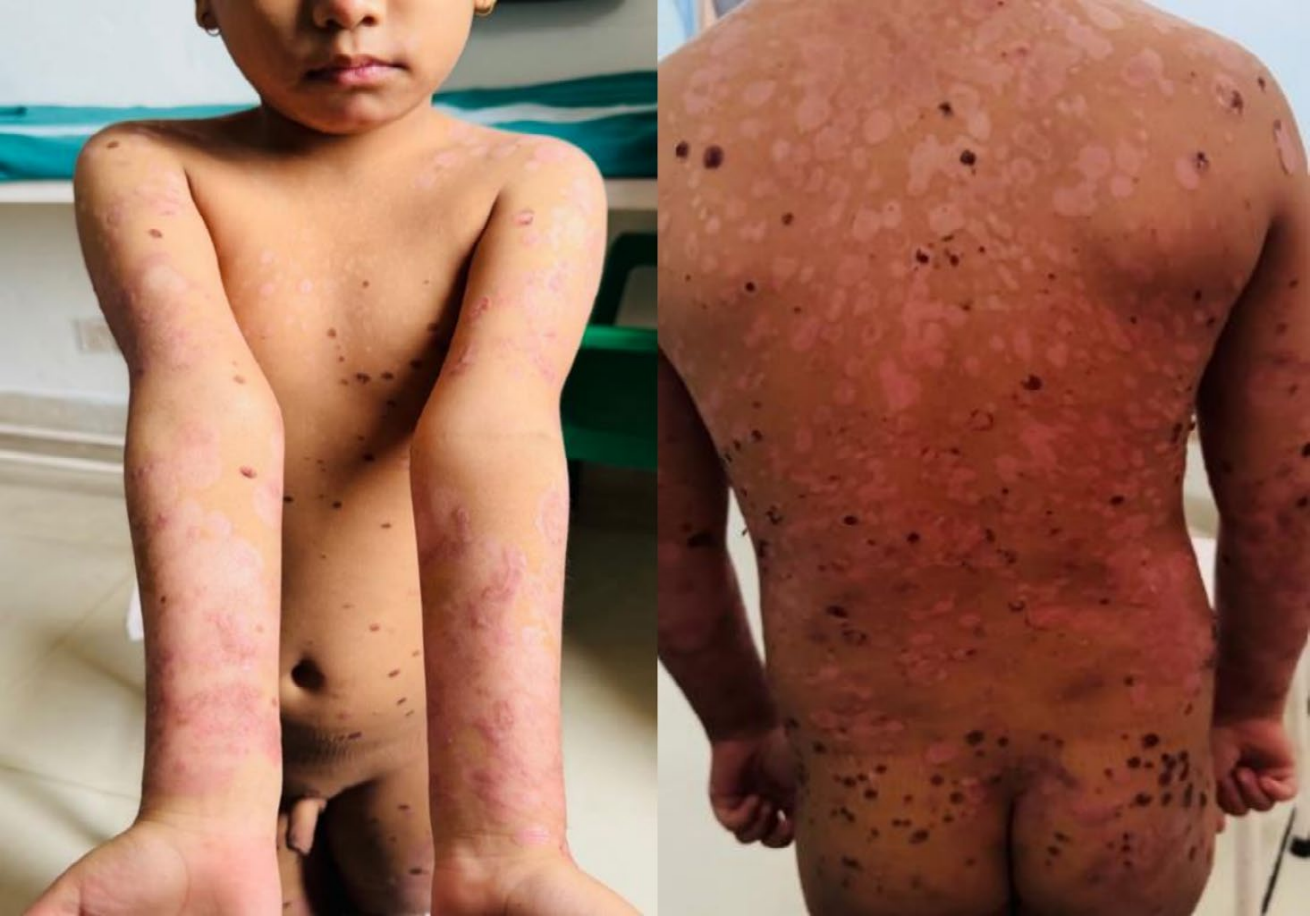
Improvement of lesions leaving post inflammatory hypopigmentation at 2‐week follow‐up.

The child was followed up at 2 weeks, which showed improvement in the lesions with post inflammatory hypopigmentation, as shown in Figure 3.

## 5 Discussion

There are wide varieties of causes of bullous eruptions in children. Infections like bullous impetigo and Staphylococcal scalded skin syndrome (SSSS) and varicella are common causes of bullous eruption. Genetic blistering diseases and autoimmune bullous disorders are uncommon causes of bullous eruptions in children, which makes the diagnosis and management of such bullous disorders challenging [5].

Chronic bullous disease of childhood (CBDC) is the most common autoimmune blistering disorder in children. There are immune deposits of immunoglobulin A (IgA) at the basement membrane zone (BMZ) of skin or mucous membranes. Target antigens like BP180 and less frequently BP230 have been identified for the production of autoantibodies [6, 7]. CBDC is often idiopathic and antibody production may be triggered by infections, drugs, vaccinations, ultraviolet radiation, malignancy or autoimmune diseases [1, 8].

CBDC generally presents in children of age between 6 months to 10 years with the mean age of 4.5 years [9]. Siegfried et al. had reported two female children aged 5.5 years and 9 years who were diagnosed as CBDC [10]. A recent case report from Nepal (2022) also demonstrated CBDC in a 5‐year‐old male child [11]. The disease presents with vesicles and tense bulla in an annular configuration over erythematous skin often described as “crown of jewels” or “string of pearls.” Lesions are usually distributed on the limbs, lower abdomen, gluteal region, and face [12]. The child in the present case also had a similar clinical presentation as described above. About half of the cases also have mucosal involvement, but we did not notice any mucosal involvement in our case. Histopathological examination in CBDC shows sub‐epidermal bullae with predominantly neutrophilic infiltrates below the basement membrane [13, 14]. Direct immunofluorescence from the perilesional skin of the back demonstrates linear deposits of IgA and C3 at the dermo‐epidermal junction, which was also seen in our case [15].

The common differentials of CBDC are bullous pemphigoid, dermatitis herpetiformis, bullous impetigo, varicella and Stevens‐Johnson syndrome/toxic epidermal necrolysis (SJS/TEN) [11, 16]. It is important to differentiate CBDC from the above conditions so that one can diagnose CBDC and initiate appropriate treatment. The drug of choice for the treatment of CBDC is oral dapsone (0.5–2 mg/kg/day) for 6 months to 1 year. Lesions begin showing improvement within 2 to 3 days of the initiation of dapsone. In our case, the child showed improvement on day‐5 of initiation of dapsone. Screening for G6PD deficiency was considered in our case before starting dapsone therapy as it can cause severe hemolysis in G6PD deficient individual which can be life threatening.

Dapsone is generally sufficient as monotherapy. It can be combined with oral steroids in severe and unresponsive cases. Other drugs that have also been used for treating skin lesions of CBDC are sulfapyridine, sulfasalazine, and sulfamethoxypyridazine. Although it is a non‐infective disorder, antibiotics like erythromycin, co‐trimoxazole, and dicloxacillin have also been found to be useful in a few cases. In children, the disease tends to spontaneously remit within 2 to 4 years after the onset [7, 17].

## 6 Conclusion

Linear IgA bullous disease of childhood or CBDC is an important immuno‐bullous disease having a similar clinical presentation to the commonly encountered bullous eruptions in children, which may lead to diagnostic confusion and delay in initiating treatment. Unavailability of immunofluorescence assay and G6PD screening facilities in peripheral hospitals of low and middle income countries (LMICs) may be an important limitation for the diagnosis and initiation of therapy in children with CBDC. Proper clinical diagnosis supported by histological and direct immunofluorescence examination helps us to diagnose CBDC and initiate proper treatment.

## Author Contributions

**Rajeev Yadav:** conceptualization, investigation, supervision, writing – original draft, writing – review and editing. **Indira Poudel:** conceptualization, investigation, software, writing – original draft, writing – review and editing. **Yogesh Poudyal:** conceptualization, supervision, writing – original draft, writing – review and editing. **Nagendra Chaudhary:** conceptualization, resources, supervision, writing – original draft, writing – review and editing.

## Consent

Written informed consent was obtained from the patient for publication of this case report.

## Conflicts of Interest

The authors declare no conflicts of interest.

## Data Availability

The data regarding this case are available with the authors and can be provided on request.
